# Real-Time Recursive Fingerprint Radio Map Creation Algorithm Combining Wi-Fi and Geomagnetism

**DOI:** 10.3390/s18103390

**Published:** 2018-10-10

**Authors:** Ju-Hyeon Seong, Dong-Hoan Seo

**Affiliations:** 1 Department of Electrical and Electronics Engineering, Korea Maritime and Ocean University, #727 Taejong-Ro, Youngdo-Gu, Busan 49112, Korea; jhseong@kmou.ac.kr; 2 Division of Electronics and Electrical Information Engineering, Korea Maritime and Ocean University, #727 Taejong-Ro, Youngdo-Gu, Busan 49112, Korea

**Keywords:** fingerprint, radio map, Wi-Fi, geomagnetism, minimum description length principle (MDLP)

## Abstract

Fingerprint is a typical indoor-positioning algorithm, which measures the strength of wireless signals and creates a radio map. Using this radio map, the position is estimated through comparisons with the received signal strength measured in real-time. The radio map has a direct effect on the positioning performance; therefore, it should be designed accurately and managed efficiently, according to the type of wireless signal, amount of space, and wireless-signal density. This paper proposes a real-time recursive radio map creation algorithm that combines Wi-Fi and geomagnetism. The proposed method automatically recreates the radio map using geomagnetic radio-map dual processing (GRDP), which reduces the time required to create it. It also reduces the size of the radio map by actively optimizing its dimensions using an entropy-based minimum description length principle (MDLP) method. Experimental results in an actual building show that the proposed system exhibits similar map creation time as a system using a Wi-Fi–based radio map. Geomagnetic radio maps exhibiting over 80% positioning accuracy were created, and the dimensions of the radio map that combined the two signals were found to be reduced by 23.81%, compared to the initially prepared radio map. The dimensions vary according to the wireless signal state, and are automatically reduced in different environments.

## 1. Introduction

Unlike Time of Flight (TOF)-based ultra-wide band (UWB) and chirp spread spectrum (CSS), which measure signal arrival times, fingerprint is an indoor-positioning technology based on measuring the received signal strength (RSS). Indoor spaces where it can be used are gradually expanding, owing to the increase in the use of wireless local area networks (WLANs) and the Internet of Things (IoT). Fingerprint can be divided into two types of technologies. One is node-based localization, which is based on indoor wireless communication technologies such as Wi-Fi [1,2,3], Bluetooth Low-Energy (BLE) [4,5,6], and Zigbee [7,8]. It uses the RSS indicator (RSSI) to show the wireless signal strength according to the presence of a node. Additionally, in order to improve the positioning accuracy, it fuses with many MEMS sensors [9] including accelerometer, gyroscope, etc. The other one is non–node-based localization, which is based on geomagnetic strength [10,11,12] and does not need a separate transmitter/receiver. The signals can be received and collected easily by mobile devices used in daily life, such as smart phones, notebook computers, and smart watches, which makes it easy to provide services directly to users. Fingerprint is divided into a training phase, in which the signal strengths are collected and used to create a radio map, and a positioning phase, in which the user position is estimated through a comparison of the radio map and the signals measured in real-time.

In order to collect the signal strengths, fingerprint requires a radio map, which is a database of the signal strengths measured based on reference points that appear at fixed position intervals on a 2D map. Unlike the time of arrival (TOA) method, which measures the arrival time of radio waves, this method has almost no need to consider the spatial structure, radio wave distortion, non-line of sight (NLOS) parameters, etc. However, the positioning resolution can become low, depending on the reference point intervals. Moreover, the process of creating the radio map requires considerable time and cost. Due to this, many algorithms [13,14,15] that combine this method with the acceleration sensors of smart devices are being developed to improve accuracy. However, in these algorithms, it must be possible to estimate a starting position. Therefore, they rely solely upon fingerprint positioning to find the user’s initial position. To improve accuracy, many studies are being conducted on fingerprint techniques that combine geomagnetism, which experiences almost no signal change over time, and Wi-Fi, which is popular and widely available [16,17,18].

Liu et al. [19] proposed an indoor control system using BLE beacon and IoTs technology. In the system, fingerprint matching based on RSSI stochastic characteristics and a posture recognition model based on geomagnetic sensing are used to establish a more efficient equipment control system, combined with Pedestrian Dead Reckoning (PDR) technology to improve the accuracy of location.

Wang et al. [20] proposed a positioning algorithm based on a particle filter, which used a radio map based on a covariance interpolation algorithm that combined Wi-Fi and geomagnetism. This positioning algorithm improved the position accuracy by effectively reducing the interference due to changes in geomagnetism. However, it required a gyroscope and acceleration sensor to perform geomagnetic matching, and the amplitude of the geomagnetism varied according to the sensor angle. Therefore, the algorithm had limitations regarding the measurement direction. Moreover, the radio map per unit space was very large, and it used a probability-based particle algorithm that required a large amount of computation, which made its processing considerably slower than that of other algorithms for the same space.

Nguyen et al. [21] proposed a method that used Wi-Fi and geomagnetic signals, and designed a radio map based on each user’s routine. This method made quick prediction possible by using the main movement paths of each user. However, it had the disadvantage that it had to store each user’s path information, which increased the size of the radio map rapidly in dense regions.

Fingerprint algorithms based on geomagnetism are very sensitive to changes in sensor angle, and it is difficult to predict signals. Therefore, it is necessary to design a radio map according to position and angle. To implement this, a vast amount of radio map measurement data is required, which rapidly increases the amount of time and workload spent in taking measurements. To overcome these problems, this paper proposes a new real-time recursive radio map creation algorithm based on Wi-Fi and geomagnetism. The proposed algorithm automatically creates geomagnetic radio maps by using geomagnetic radio-map dual processing (GRDP) in the training phase, to remove the existing radio map creation processes involving geomagnetic methods that require measurements for each angle and consume a lot of time. Based on this phase, in the positioning phase, the proposed MDLP-based radio map updating greatly reduces the sizes of the radio maps by actively optimizing their dimensions using the entropy-based minimum description length principle (MDLP) method, rather than the support vector machine (SVM) method that simply classifies the RSSI.

## 2. Related Theories

### 2.1. Geomagnetism-Based Fingerprint

**Figure 1:**
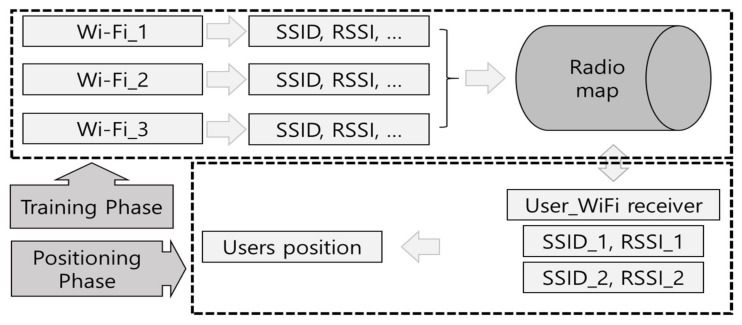
Typical fingerprint flow chart.

A typical flowchart for fingerprint, showing both the training phase and positioning phase, regardless of the signal type, is presented in Figure 1. The training phase measures the signal strength at reference points and creates a radio map. The signal information received from a single Wi-Fi source includes a variety of information, including the service set identifier (SSID), RSSI, and link quality indicator (LQI). Of these, the SSID, which can distinguish each access point (AP), and the RSSI, which indicates the relative signal strength, are necessary for finding the position [22]. The Wi-Fi signals around a given position (reference point) are measured. Several APs are explored, and the Wi-Fi signal characteristics at these positions are stored, forming a radio map. During the positioning phase, this radio map is used to find the user’s positions in real-time. To improve the accuracy of the radio map, the signal at each reference point is measured several times and the radio map is created from the average value. When probability-based positioning algorithms are used, the created radio map additionally includes the variance and the maximum and minimum values of the signal strength. The radio map’s structure is generally similar to

(1) $${\mathrm{RM}}_{RP}=\left[\begin{array}{ccccc} P_{AP1\left(1\right)} & P_{\mathrm{AP}1\left(2\right)} & P_{\mathrm{AP}1\left(3\right)} & \cdots & P_{\mathrm{AP}1\left(n\right)}\\ P_{AP2\left(1\right)} & P_{\mathrm{AP}2\left(2\right)} & P_{\mathrm{AP}2\left(3\right)} & \cdots & P_{\mathrm{AP}2\left(n\right)}\\ P_{\mathrm{AP}3\left(1\right)} & P_{\mathrm{AP}3\left(2\right)} & P_{\mathrm{AP}3\left(3\right)} & \cdots & P_{\mathrm{AP}3\left(n\right)}\\ \vdots & \vdots & \vdots & \cdots & \vdots \\ P_{\mathrm{APk}\left(1\right)} & P_{\mathrm{APk}\left(2\right)} & P_{\mathrm{APk}\left(3\right)} & \cdots & P_{\mathrm{APk}\left(n\right)} \end{array}\right]$$

where ${\mathrm{RM}}_{RP}$ is the set of all collected RSSI data (radio map). $P_{\mathrm{APk}\left(n\right)}$ is the RSSI measured at n, which is the *k*th reference point from the AP. Generally, the RSSI is between −30 and −100 dBm. As the relative positions of the node and user become farther apart, the signal strength approaches −100 dBm. Based on the RSSI measured from each node at the reference point, the positioning phase measures the position using a probability model [2,23], learning algorithm [24], etc., which can compare the radio map in Equation (1) and the RSSI measured in the actual space in real time. 

However, geomagnetic fields have directionality, and nodes do not exist separately; therefore, the structure of the radio map should be designed differently. Geomagnetic fields are measured at the level of several μT, and to measure them, geomagnetic sensors measure three signal strengths, in the *x*, *y*, and *z* directions. However, if the radio map is created leaving the data of each axis as it is, the amount of data becomes vast, and the position accuracy may be reduced owing to the signal distortion caused by the drift phenomena in the signals acquired from each axis and the changes in the sensor angle. Offset, which is the sensor’s internal error, misalignment between axes, and the sensitivity of each axis are inherent sensor errors, and there are limits to overcoming them. 

**Figure 2:**
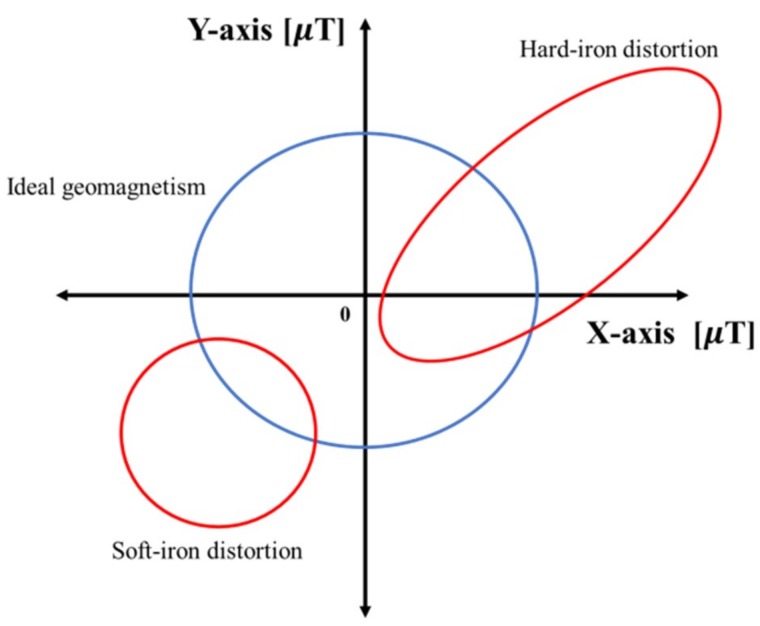
Ideal geomagnetic characteristics and geomagnetic characteristics due to distortion.

**Figure 3:**
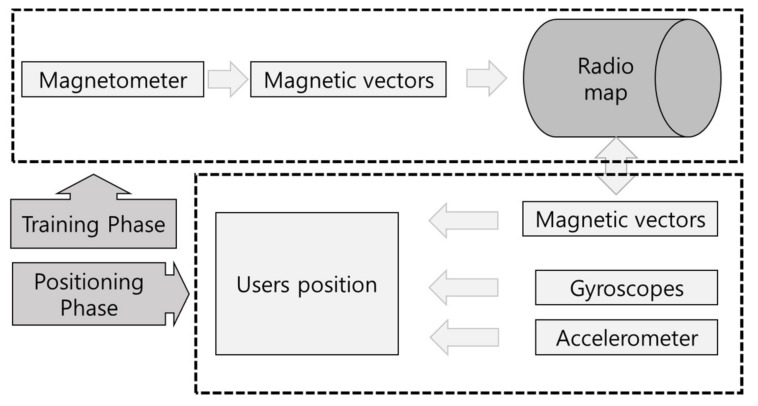
Geomagnetic based fingerprint flow chart.

Currently, many studies are being conducted on overcoming hard-iron distortion and soft-iron distortion, which are external distortion factors [25,26]. However, these methods revise the characteristics of the magnetic field through post-processing; therefore, it is difficult to use them for positioning, which requires real-time processing. An ideal signal without distortion creates a spherical shape, similar to the blue sphere in Figure 2. However, when distortion occurs, the earth’s magnetic field is measured as an ellipse, similar to the red shape. The geomagnetic-based fingerprint is shown in Figure 3. Generally, many studies have been made by integrating the gyroscope and accelerometer in order to correct the accuracy of the geomagnetic. Additionally, the radio map is adapted in various ways, such as storing the *x*, *y*, and *z*-axis signals themselves or storing geomagnetic field vectors. When converting geomagnetic values to vectors, an often-used method [27] assumes the height to be fixed as in Equation (2) and calculates the *x*, *y*, and *z*-axis signals in order to minimize the radio map.

(2) $$\;GA_{i}=\sqrt{G_{xi}^{2}+G_{yi}^{2}+G_{zi}^{2}}\;$$

Here, $GA_{i}$ is geomagnetic field vector at position i. $G_{xi}$ and $G_{yi}$ are the strengths of the *x*, *y*, and *z*-axis components at i. The set of vector intensity calculated in this manner constitutes the radio map, and is stored as shown in Equation (3).

(3) $$\;{\mathrm{RM}}_{G}=\left[\begin{array}{c} GA_{1}\\ GA_{2}\\ GA_{3}\\ \vdots \\ GA_{i} \end{array}\right]\;$$

However, in this method, if the measurement angle is not fixed, these intensities of geomagnetic vector change in a way that is not fixed, owing to distortion. Therefore, highly reliable position accuracy can only be ensured if fixed angles are maintained; however, there are difficulties in performing real-time processing. Therefore, a method [19] that creates a radio map according to the changes in the angles are being used. The positioning accuracies of these methods are very high, with regard to angle changes, but the radio maps become very large; therefore, their processing speed is slow.

### 2.2. Minimum Description Length Principle

The minimum description length principle (MDLP) is often used in machine learning and probabilistic modeling, and it is a typical discretization technique that uses class entropy to represent the degree of disorder in continuous data. The entropy has a value between 0 and 1, depending on the continuous data set’s correlation and continuity, and is expressed as shown in Equations (4) and (5) below.

(4) $$Ent\left(S\right)=-\sum_{j=1}^{k}P\left(C_{j},S\right)\log\left(P\left(C_{j},S\right)\right)\;$$

(5) $$\;P\left(C_{j},S\right)=\;\frac{Count\left(C_{j},S\right)}{\left|S\right|}\;$$

Here, S is the set of given data. $C_{j}$ is the set of class values. $Count\left(C_{j},S\right)$ is the number of classes in $C_{j}$ in *S*. |S| shows the total number of given data. If a single set of continuous signals approaches an entropy value of 0 via the equation above, it means that the data have a single fixed value and that this set (or signal) is highly reliable. On the other hand, if the entropy approaches 1, it means that the signal is insufficiently fixed and has low reliability. Therefore, MDLP has the benefit of simplifying and optimizing the data in complex circumstances with a large amount of data. Based on Equations (4) and (5), MDLP is expressed as in Equation (6) below.

(6) $$\;E\left(X,T,S\right)=\frac{\left|S_{1}\right|}{\left|S\right|}Ent\left(\left|S_{1}\right|\right)+\frac{\left|S_{2}\right|}{\left|S\right|}Ent\left(\left|S_{2}\right|\right)\;$$

Here, X is the number of continuous variables. S is the data set. T is the split point that divides set *S* into sets $S_{1}$ and $S_{2}$. If it is calculated recursively according to the conditions restricted by the user, set *S* can be divided to the desired extent. If the value found from these results approaches 0, it means that there is almost no regularity in the continuous signal. This means that making clear distinctions is difficult, and thus, division is impossible.

## 3. Proposed Radio Map Description

### 3.1. Proposed Radio Map Based on Geomagnetic Field and Wi-Fi

**Figure 4:**
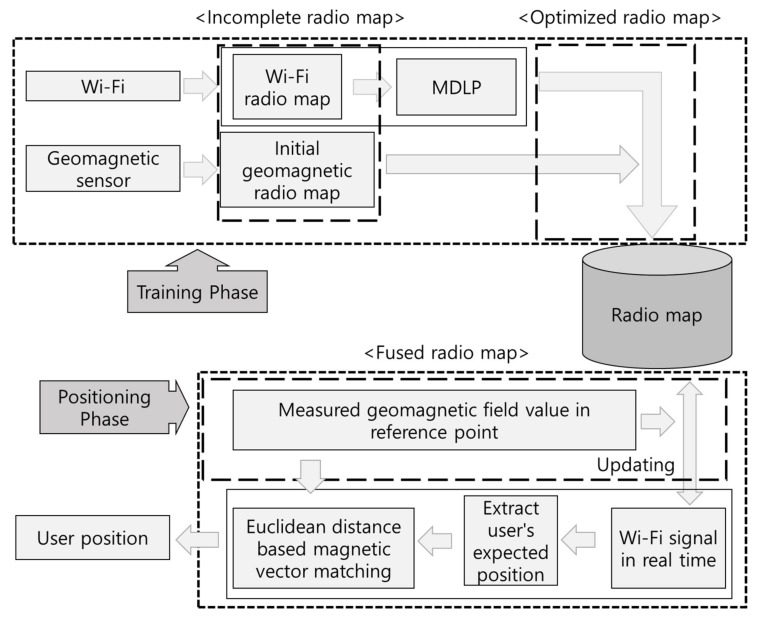
Proposed radio map structure based on Wi-Fi and geomagnetism.

Figure 4 shows the structure of the proposed real-time recursive radio map based on Wi-Fi and geomagnetism. The initial geomagnetic radio map created is vulnerable to changes in the direction of geomagnetism because the geomagnetic signals, which are collected at the same time as the Wi-Fi signals, have intensities of the geomagnetic vector in one direction only. In the case of the Wi-Fi radio map collected at this time, all Wi-Fi signals are collected from within the space where positioning is to be performed. These signals can be classified and removed via MDLP, and the size of the map can be optimized actively according to the disorder of the signals received from the node, to create an optimized radio map. Subsequently, if the Wi-Fi radio map and the initial geomagnetic radio map are combined into a single radio map, the incomplete geomagnetic radio map of the reference points estimated on the radio map can be supplemented with updates. Based on the expected position of the user due to Wi-Fi signal matching, we finally determine the location of the user by comparing the geomagnetic vectors again. The combined Wi-Fi and geomagnetic radio map created by the updates have improved user-positioning accuracy and can perform positioning in emergency situations such as blackouts.

### 3.2. Fusion Radio Map Data Acquisition

**Figure 5:**
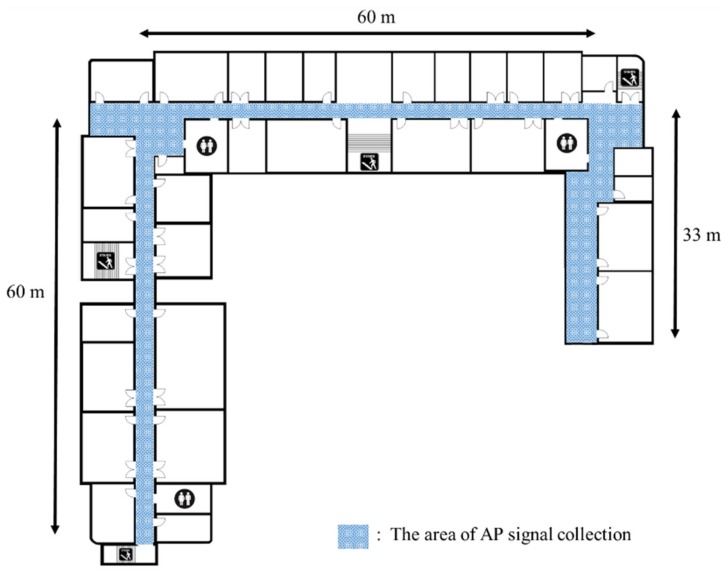
Experiment space to adapt the proposed algorithm.

The radio map is used as the reference position that estimates the user’s position in the positioning phase. Therefore, radio map creation is a very important process, which can determine the accuracy of the positioning system. To confirm the proposed algorithm’s performance, a 150 m section of a hallway was used as the experiment area, as shown in Figure 5. The experiment area was divided into a 120 m section in which sufficient Wi-Fi signals could be collected, and a 30 m section with almost no Wi-Fi receivers, to create a suitable environment for testing the proposed algorithm’s performance. To collect the signals, a C#-based signal-collection program, which could measure the Wi-Fi SSID and RSS in real-time was created. The Wi-Fi network measures the public and private Wi-Fi APs used in the building as they are, and a radio map of that location is created simultaneously as the measurement is completed at one reference point. The sampling times of Wi-Fi and geomagnetic signals are set to 1 s and 0.1 s, respectively. The measurement time of the signal is set to 10 s for each reference point, and the Wi-Fi signal measurement and the geomagnetic signal for one azimuth are measured at the same time. To minimize the change of the state of the AP as well as physical influences (opening/closing of doors, presence of obstacles, etc.), the settings of the used AP, such as antenna characteristics, the number of antennas, and the position, were maintained. In Wi-Fi signals, the deviation of the measured RSSI is relatively large, even in the same position, owing to the receiving/transmitting environment. Therefore, reference points were set within 3 m intervals, over which signal identification was possible, to accurately distinguish the signals. The data were collected at a fingerprint radio-map’s normal signal acquisition interval of 3 m, to acquire the Wi-Fi and geomagnetic signals. There were 53 reference points and 43 measured nodes in the experiment space, and signals were gathered in a walking survey, for accuracy.

**Figure 6:**
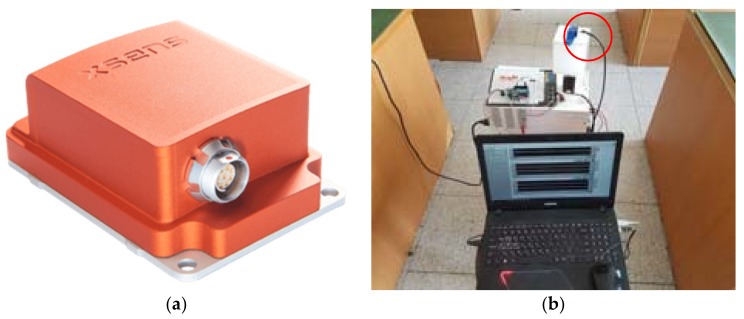
Wi-Fi and geomagnetic signal collection positions and structure for creating the radio map: (**a**) Geomagnetic sensor for experiment; (**b**) experimental system for collecting the geomagnetic and Wi-Fi signals.

Figure 6 shows the measurement equipment used to collect the Wi-Fi and geomagnetic signals. In Figure 6a, an Xsense MTi-10 was used as the geomagnetic measurement sensor, and the Wi-Fi signal was collected by an Android-based app; Figure 6b shows the experimental system used for collecting the geomagnetic and Wi-Fi signals. The red circle represents the geomagnetic sensor, and in order to obtain geomagnetic data according to the change of angle, the center of the sensor was fixed as the center axis of rotation. For radio map management and optimization, MySQL was used, and a separate server was built. This server was connected to a smartphone so that the radio map could be updated and optimized in real time.

### 3.3. Creating and Updating the Proposed Radio Map

The initial Wi-Fi and geomagnetic signals, which were measured to create the radio map, appear in the form of Equations (1) and (3), respectively. The Wi-Fi signal’s matrix size is 139 × 53 (number of nodes × number of reference points), and the geomagnetic signal’s matrix size is 1 × 53. The matrix of the geomagnetic signal measured at this time does not consider all azimuths, but represents the strengths of the geomagnetic signals measured at the reference points. MDLP is applied to the initial radio map, which is based on these signals, and the data are automatically optimized. The structures of the initial radio map and optimized radio map are as shown below, in Equation (7).

(7) $${\mathrm{RM}}_{GW}=\left[\begin{array}{ccccc} P_{\mathrm{AP}1\left(1\right)} & P_{\mathrm{AP}1\left(2\right)} & P_{\mathrm{AP}1\left(3\right)} & \cdots & P_{\mathrm{AP}1\left(53\right)}\\ P_{\mathrm{AP}2\left(1\right)} & P_{\mathrm{AP}2\left(2\right)} & P_{\mathrm{AP}2\left(3\right)} & \cdots & P_{\mathrm{AP}2\left(53\right)}\\ P_{\mathrm{AP}3\left(1\right)} & P_{\mathrm{AP}3\left(2\right)} & P_{\mathrm{AP}3\left(3\right)} & \cdots & P_{\mathrm{AP}3\left(53\right)}\\ \vdots & \vdots & \vdots & \cdots & \vdots \\ P_{\mathrm{APk}\left(1\right)} & P_{\mathrm{APk}\left(2\right)} & P_{\mathrm{APk}\left(3\right)} & \cdots & P_{\mathrm{APk}\left(53\right)}\\ GA_{1\;} & \;GA_{2} & \;GA_{3}\; & \cdots & GA_{53} \end{array}\right]$$

The structures of the initial radio map and optimized radio map are the same, but their dimensions are reduced while maintaining the initial measured geomagnetic values; therefore, the Wi-Fi radio map is also reduced. The Wi-Fi RSSIs (P) and the 53 geomagnetic sensor values (GA) obtained from the 53 reference points in the experiment environment form the basic radio map for positioning. Based on this optimized radio map, the positioning phase performs updating and positioning simultaneously, to create the geomagnetic radio map. User positioning is possible through Wi-Fi, which is not affected by azimuths. Thus, it is possible to measure the geomagnetic strengths of the reference points simultaneously based on the Wi-Fi positions. As this is being updated, the geomagnetic values cannot be unconditionally updated because of the continuous increases in the radio map. Therefore, the number of updates according to the reference points is limited.

**Figure 7:**
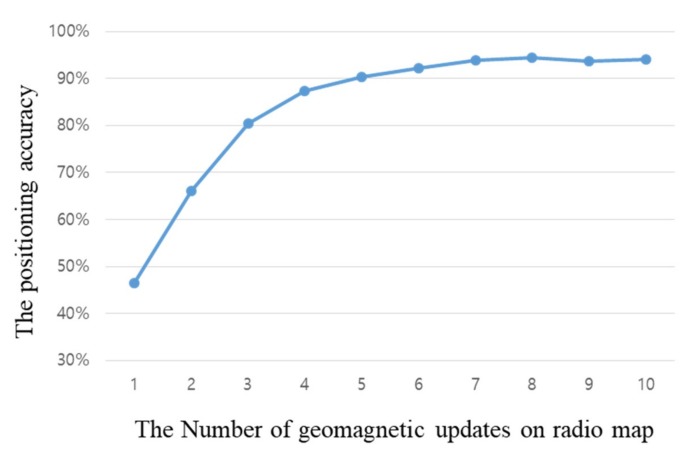
Positioning accuracy according to the number of geomagnetic sensor updates.

Figure 7 shows the accuracy according to the number of times the radio map was updated with geomagnetic sensors alone, having removed the Wi-Fi radio map. It can be seen that the accuracy improves with an increase in the number of geomagnetic updates; however, after eight updates, there is almost no change in the accuracy. This means that, as the geomagnetic signal strength alone is recreated in the radio map, sections with similar signals must have occurred, which makes it difficult to improve the accuracy further. Based on the experiment results, the number of updates is limited to eight. For signals measured after that, whether or not an update should occur is decided through comparisons with already created geomagnetic signals. The continuity of signals related to the geomagnetism’s azimuth is not evident; therefore, deciding whether updates should occur using data features is difficult. When signals that are similar are found during a comparison of the signals collected according to the user’s movement position and real-time geomagnetic signals, the cumulative mean value is used to gradually stabilize the radio map according to the amount of usage. If a magnetic field vector with a new intensity is measured, the similarity is confirmed based on its Euclidean distance with the intensities of the magnetic field vector from adjacent reference points, and that the position’s geomagnetic vector update is performed to minimize update position errors. Finally, we apply the Euclidean distance to the Wi-Fi signal, as shown in Equation (8), to estimate the location of the user based on the proposed radio map.

(8) $$Dist\left(i\right)=\sqrt{\sum_{j=1}^{n}{\left({\mathrm{AP}}_{j}-{\mathrm{AP}}_{rj}\right)}^{2}}\;$$

where ${\mathrm{AP}}_{j}$ is the RSSI of the AP with the same SSID (j) in the radio map, and ${\mathrm{AP}}_{rj}$ is the RSSI of the same SSID measured in real time. Through this equation, three reference points having the smallest value are extracted as the positions of the potential users. Then, we apply the Euclidean distance again to the total of 24 geomagnetic intensities stored in the radio map at three reference points, and the current position of the user is derived from the reference point with the smallest difference in value.

## 4. Experiments and Evaluation

**Figure 8:**
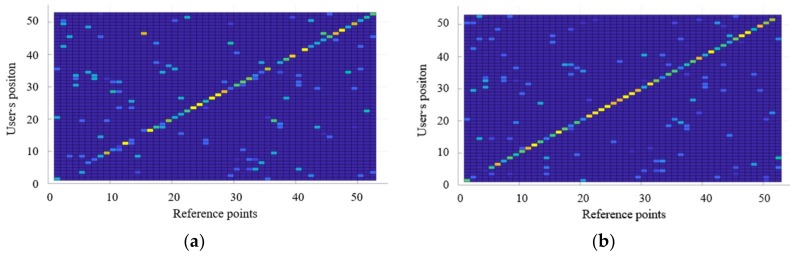

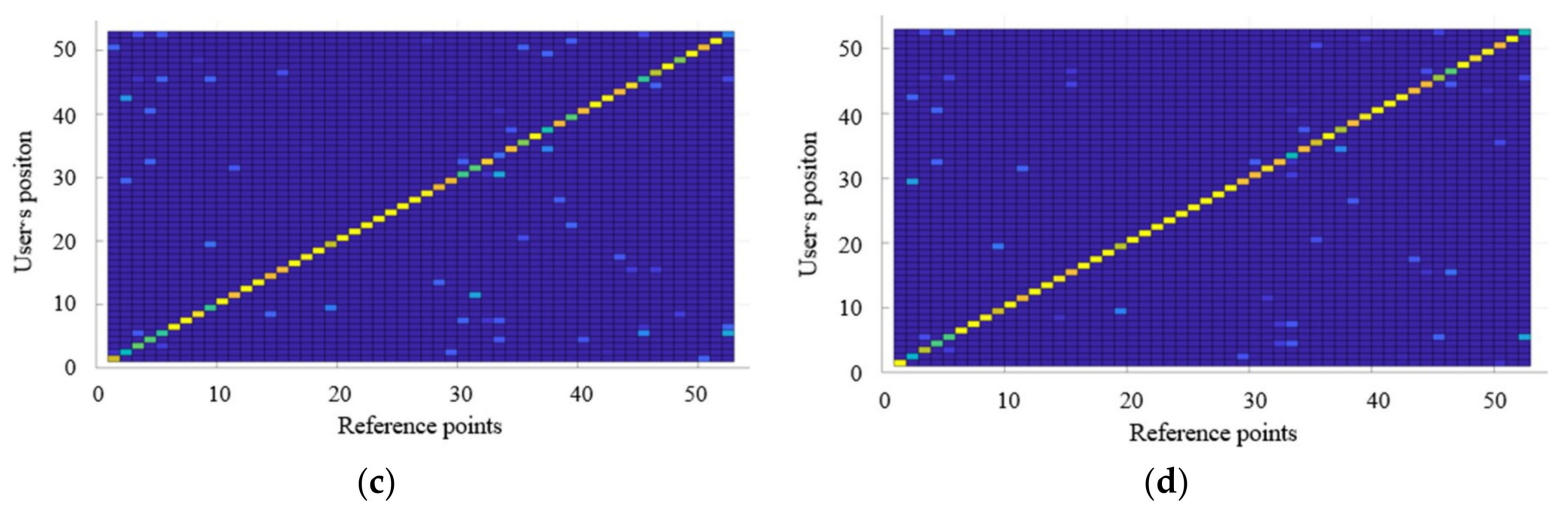
Analysis of positioning accuracy of the proposed radio map, according to the number of geomagnetic signal acquisitions: (**a**) Initial radio map (one acquisition); (**b**) three acquisitions; (**c**) six acquisitions; (**d**) positioning accuracy after applying the proposed algorithm (eight acquisitions).

To verify the proposed algorithm, experiments were performed based on 53 reference points set in the experimental environment. Figure 8 shows the accuracy of the proposed positioning method, according to the number of geomagnetic signal acquisitions. The *x*-axis represents the 53 reference points and the *y*-axis represents the position estimation results. To express all the positions estimated according to the reference points, the results are shown through a confusion matrix. The clearer the diagonal line representing the same values in the *x* and *y*-axes is, the higher the positioning accuracy will be. As the accuracy of positioning increases, the display color becomes closer to yellow, and as the number decreases, the display color becomes closer to blue.

Figure 8a shows the position estimation results of using an incomplete radio map of the geomagnetic signals collected simultaneously with Wi-Fi during data collection. The incomplete radio map has low positioning accuracy because it stores the geomagnetic signals for one random direction only. However, as the position recognition is performed more times, the positioning accuracy improves gradually, as shown in Figure 8b–d, where the geomagnetic signals are collected three, six, and eight times, respectively. The experimental results confirm that the positioning accuracy of the final result of the proposed radio map in Figure 8d is improved by 88% compared to that using an incomplete radio map (Figure 8a). As the geomagnetic field vectors can be collected and constructed simultaneously through the Wi-Fi fingerprint, without the need for collecting them from different directions, a radio map of the two communications of Wi-Fi and geomagnetism can be constructed and updated with no separate processes, and the recognition performance can be improved through mutual complementation of positions.

Table 1 shows a comparison of the creation time of the radio map in the training phase with the same sampling time. Since the generation time of the radio map varies depending on the measurement environment, such as the space and the AP state, the proposed algorithm sets the measurement time at one reference point to 1 unit. As the geomagnetism-based radio map has different signal magnitudes depending on the angle, it is necessary to measure it according to the angle. Therefore, based on the proposed algorithm, the geomagnetism-based radio map requires 8 times of iterations, depending on the angle. On the other hand, the proposed algorithm creates the radio map after post-processing in the positioning phase, so it is measured at the same time as the existing Wi-Fi based fingerprint.

**Figure 9:**
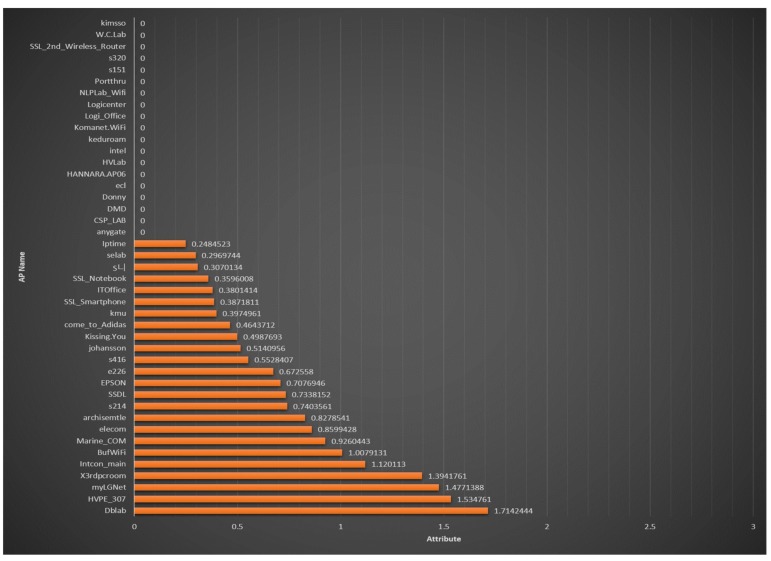
Positioning accuracy according to the number of geomagnetic sensor updates.

Figure 9 shows the results of the proposed MDLP-based radio-map optimization in the training phase. The *x*-axis shows the calculated MDLP values and the *y*-axis shows the SSIDs of the nodes (Wi-Fi APs). Of the above 43 Wi-Fi signals, 19 nodes show a value of 0, and will be removed automatically. If a node has a value exceeding 1, it means that the RSSI is clearly distinguished according to the reference point, and that it is a highly reliable node. This MDLP method has the advantage that it can be used when the indoor environment changes, and it can be used with any RSSI-based fingerprint algorithm, such as Zigbee, BLE, etc.

Table 2 shows a size comparison of the dimensions of the initially created radio map, the radio map that was reduced and optimized by MDLP, and the radio map that was ultimately created by GRDP. The initial radio map was created using 43 Wi-Fi signals collected from 53 reference points. Using MDLP, the signals with poor distinction according to the reference point were removed, and the map’s dimension was reduced to 24. Based on this, the geomagnetic signals were updated in the positioning phase and used to create a dimension of 32. The number of wireless signals removed by the optimization process can vary according to the surrounding environment, including factors such as the Wi-Fi signal state, spatial structure, and conditions of the medium. Only the state of the signal is considered; therefore, the method can be used in any environment. If there is an indoor space with a complex structure and a definite signal distinction according to the reference point, the signal can be optimized more definitely to improve the positioning performance.

**Figure 10:**
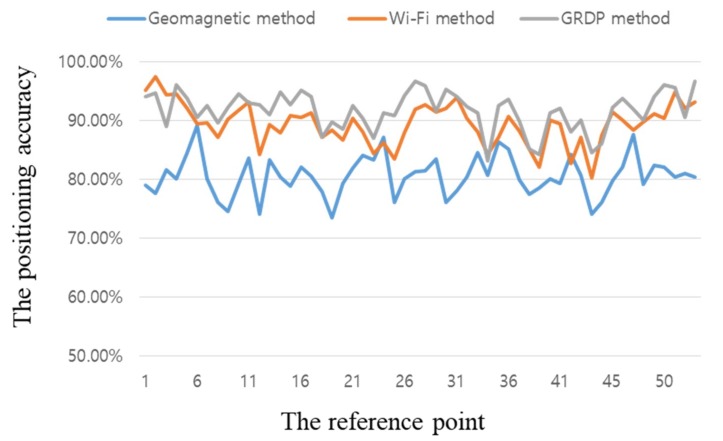
Positioning accuracy according to the proposed algorithm’s reference points.

Figure 10 shows the performance of the radio map created via the proposed algorithm. The *x*-axis shows the reference points and the *y*-axis shows the resultant positioning accuracy. The average geomagnetic positioning performance of the radio map created by collecting geomagnetic signals eight times was 91.72%. If only the geomagnetic method is applied, it can be seen that the overall accuracy is very low. However, since the proposed algorithm reduces the range of the position estimation by Wi-Fi, it can improve the position accuracy by eliminating the overlapping vector intensities at all the existing positions. On average, the performance of the proposed algorithm was high, but there were slight positioning performance degradations at reference points 1–3, 34, 52, and a few others. The main reason for this was the drop in accuracy caused by disturbances in the real-time sensor measurement values. It was confirmed that the proposed algorithm improved the initial positioning performance, which was required for continuous positioning at a given location, and the geomagnetic radio map was created properly. If one intends to begin position tracking in a given indoor space, the proposed algorithm will be essential, and can be used in emergencies such as blackouts.

## 5. Conclusions

This paper proposed a new real-time recursive radio map creation algorithm based on Wi-Fi and geomagnetism. The proposed algorithm consists of GRDP and MDLP-based radio map updating to automate and minimize the workload and time consumed in creating radio maps, which are needed for fingerprint-based positioning that combines Wi-Fi, which is very widely used, and geomagnetism, which is very sensitive to changes in angle. In the training phase, the proposed GRDP combines geodetic techniques, which require measurements at each angle and are time-consuming, with the existing Wi-Fi wireless mapping process.

We also proposed an algorithm to actively optimize the radio maps by reducing their dimensions via MDLP, which automatically distinguishes and removes unnecessary Wi-Fi signals in the positioning phase. To minimize the radio map creation time and the positioning errors that occur because of the angle when positioning is performed via geomagnetism alone, a recursive algorithm was proposed, which updated the geomagnetism signal during the phase in which the actual user’s position was recognized to create a complete radio map. The results of experiments using actual measurements showed that the geomagnetic radio map was created in the same amount of time as the radio map for Wi-Fi-based positioning, and that it had a positioning performance exceeding 80%. It was reduced by 80% while the collection times of geomagnetism signals according to the direction of heading of the sensor in the training phase. Moreover, it was confirmed that the radio map was optimized through automated dimension reductions using MDLP. The proposed algorithm provides the advantages of collecting geomagnetic signals and automatically constructing a radio map of two communications, and mutually complementing the positioning performance by automatically updating the geomagnetic signals according to the movement of the user, with no separate process during the construction of the Wi-Fi-based fingerprint.

## Figures and Tables

**Table 1:** Comparison of generation time of radio map in training phase (same sampling time).

	Geomagnetic Based Radio Map	Conventional Wi-Fi Radio Map	Proposed Radio Map
Creation time	8 unit	1 unit	1 unit

**Table 2:** Changes in radio-map dimensions and positioning accuracy according to the proposed positioning phase.

	Incomplete Radio Map	Optimized Radio Map	Ultimately Proposed Radio Map
Size of radio map (number of APs in radio map)	54 × 43	54 × 24	54 × 32
Wi-Fi–based positioning accuracy (reference points-level)	82.17%	89.44%	89.44%
Geomagnetism-based positioning accuracy (reference points-level)	46.45%	46.45%	80.62%
Wi-Fi/geomagnetism combined positioning accuracy (reference points-level)	-	-	91.72%
